# A new gecko from the earliest Eocene of Dormaal, Belgium: a thermophilic element of the ‘greenhouse world’

**DOI:** 10.1098/rsos.220429

**Published:** 2022-06-29

**Authors:** Andrej Čerňanský, Juan D. Daza, Richard Smith, Aaron M. Bauer, Thierry Smith, Annelise Folie

**Affiliations:** ^1^ Department of Ecology, Laboratory of Evolutionary Biology, Faculty of Natural Sciences, Comenius University in Bratislava, Mlynská dolina, Bratislava 84215, Slovakia; ^2^ Department of Biological Sciences, Sam Houston State University, Huntsville, TX, USA; ^3^ Directorate Earth and History of Life, Royal Belgian Institute of Natural Sciences, 29 rue Vautier, B-1000, Brussels, Belgium; ^4^ Scientific Survey of Heritage, Royal Belgian Institute of Natural Sciences, 29 rue Vautier, B-1000, Brussels, Belgium; ^5^ Department of Biology and Center for Biodiversity and Ecosystem Stewardship, Villanova University, Villanova, PA 19085, USA

**Keywords:** squamata, early Palaeogene, European archipelago, Palaeocene–Eocene thermal maximum

## Abstract

We here describe a new gekkotan lizard from the earliest Eocene (MP 7) of the Dormaal locality in Belgium, from the time of the warmest global climates of the past 66 million years (Myr). This new taxon, with an age of 56 Myr, together with indeterminate gekkotan material reported from Silveirinha (Portugal, MP 7) represent the oldest Cenozoic gekkotans known from Europe. Today gekkotan lizards are distributed worldwide in mainly warm temperate to tropical areas and the new gecko from Dormaal represents a thermophilic faunal element. Given the Palaeocene–Eocene thermal maximum at that time, the distribution of this group in such northern latitudes (above 50° North – the latitude of southern England) is not surprising. Although this new gekkotan is represented only by a frontal (further, dentaries and a mandibular fragment are described here as Gekkota indet. 1 and 2—at least two gekkotan species occurred in Dormaal), it provides a new record for squamate diversity from the earliest Eocene ‘greenhouse world’. Together with the Baltic amber gekkotan *Yantarogekko balticus*, they document the northern distribution of gekkotans in Europe during the Eocene. The increase in temperature during the early Eocene led to a rise in sea level, and many areas of Eurasia were submerged. Thus, the importance of this period is magnified by understanding future global climate change.

## 1. Introduction

The fossil record of lizards in general is relatively well-known in Europe, especially from the middle and late Eocene, in part thanks to exceptional localities such as Messel in Germany [1]. However, data regarding the early Eocene are unfortunately scant. The locality of Dormaal in Belgium represents one of the rare exceptions, serving as a window to the earliest Eocene (MP 7 reference level of the mammalian biochronological scale for the European Palaeogene; BiochroM'97 [2]) ‘greenhouse world’. The early Eocene is particularly interesting because the Eocene climate began with a rapid and intense warming called the Palaeocene–Eocene thermal maximum (PETM), which is marked by the Palaeocene–Eocene carbon isotope excursion (CIE), 56 Ma. In fact, the warmest global climates of the past 66 Myr occurred during the early Eocene epoch (about 56 to 48 Ma) when megathermal floral elements, including palms, were present even in Antarctica [3,4]. However, many crucial aspects of the beginning of the Eocene as a ‘bridge’ between older Palaeocene and later Eocene periods remain unknown. The distribution, richness and diversity of squamates, as ectothermic animals, are highly dependent on temperature and climatic conditions [5–7], and these climate changes had marked impacts on their history [8]. But our understanding of lizard palaeodiversity during this important time period as a whole is very limited.

Here we report on a new gekkotan lizard from Dormaal. The occurrence of geckos at this locality was firstly mentioned by Augé ([9, p. 103]; a fragment of a right dentary is included in the list of materials allocated as ‘Gekkonidae indet’, but is not figured or described). The Dormaal fossils and those reported from Silveirinha (MP 7) in Portugal [10] represent the earliest Cenozoic representatives of gekkotans known in Europe. It should be noted that Rage and Augé [10] stated that the occurrence of this group in Dormaal was firstly reported in Augé [11]. However, this seems to be an error, because that paper deals with no gekkotans. Rather, it is written that gekkotans can be found only in the slightly younger locality of Condé-en-Brie (MP8 + 9), whence this group was described in the same year by the same author [12]. The use of Gekkonidae in these previous papers equates today to the crown group Gekkota. Gekkota is a clade comprising over 2000 extant species of geckos and pygopods [13–15]. Although some of the oldest stem-gekkotans (*Norellius* [16,17] and *Gobekko* [18]) include articulated and complete skulls, and astonishing specimens with superb preservation of skeletal and soft tissue are also reported in amber from the Cretaceous of Myanmar [19,20]; the Cenozoic fossil record of these successful and cosmopolitan lizards is generally scarce. It is represented mainly by isolated skull elements and vertebrae [9,21–24]. One exception is a well-preserved specimen in Baltic amber from the Eocene of northwestern Russia described by Bauer *et al*. [25] as *Yantarogekko balticus.* The external (surface) features of this specimen are very complete, but computed tomography (CT) scans of the specimen reveal no preserved skeletal material (Johannes Müller 2017, personal communication). The age of Baltic amber is not certain, and its accumulation was not the result of a single, long-term process during the Lutetian-Priabonian, but was repeated independently in two different stages—Bartonian and Priabonian, respectively [26]. Besides this single Baltic amber specimen, gekkotans are also present in amber from the Miocene of the Dominican Republic, where they are represented by multiple specimens referable to the extant sphaerodactyl genus *Sphaerodactylus* [27–29].

**Figure 1.**
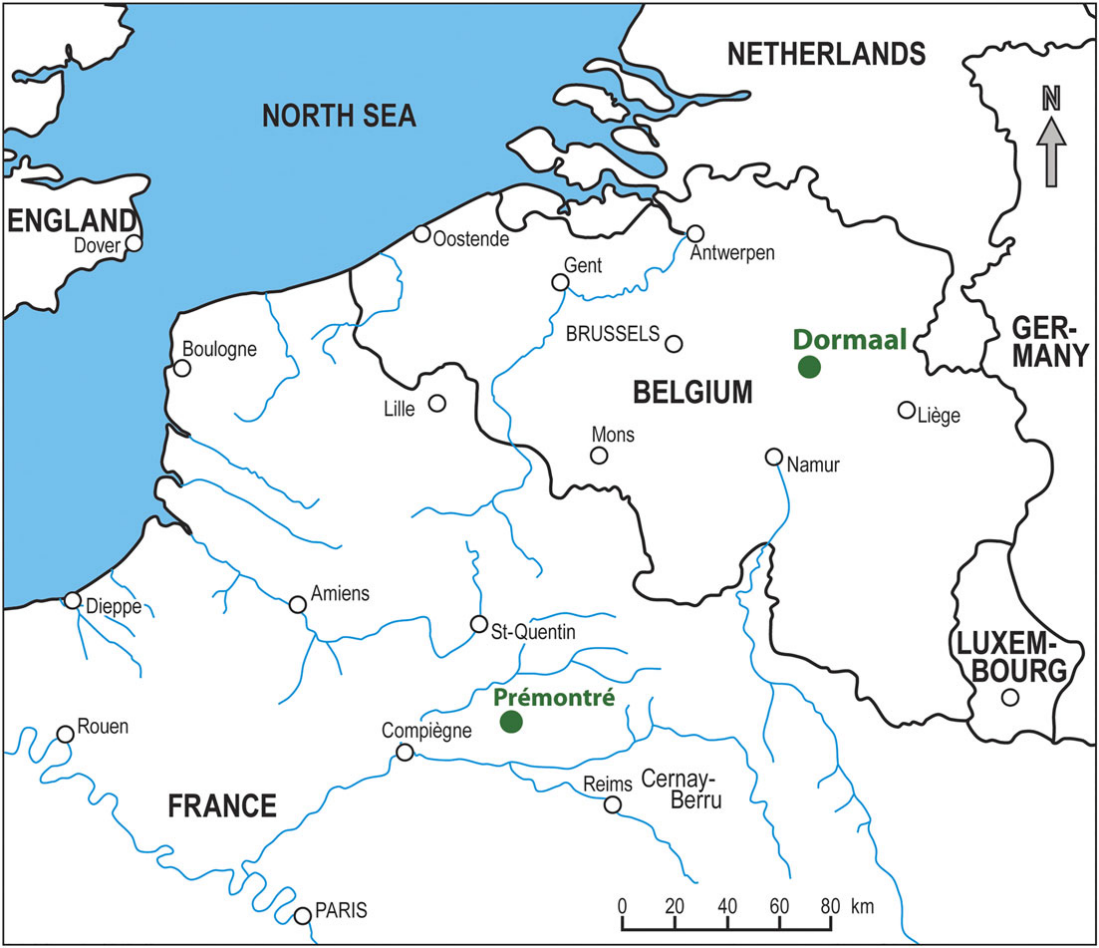
Location of the earliest Eocene locality of Dormaal (MP7, Belgium) that has yielded *Dollogekko dormaalensis* gen. et sp. nov. and the early Eocene locality of Prémontré (MP10, France) that has yielded *Laonogekko lefevrei*.

The rarity of gekkotans in the fossil record and their typical representation by only certain elements (frontal, maxilla, dentary and presacral vertebrae) most likely reflect their lightly built skeleton [30,31] and the resilience of these bones, as also reflected in their resistance to destruction by digestion of predators [32]. Moreover, these elements are highly recognizable (because they contain highly diagnostic features), whereas others are not. Unfortunately, this leads to significant gaps in our knowledge of the evolution of this clade. This is especially true for the Palaeogene members of the group [9,21,23,33,34]. The fossils from this period are mainly known from Europe, but most of the material is disarticulated, except the above-mentioned *Yantarogekko balticus*, a partly preserved, but undescribed, specimen from Messel [1] and a sphaerodactylid, *Geiseleptes delfinoi*, from Geiseltal [35]. The Palaeogene taxa include *Laonogekko lefevrei* described by Augé [36] from the early Eocene of Prémontré (MP 10, Paris Basin, France; figure 1). The material consists of isolated jaws and a frontal bone. The middle Eocene gekkotans are represented by *Rhodanogekko vireti* described by Hoffstetter [37] from Lissieu (MP 14, eastern France). This taxon is based on a single frontal. The late Eocene/early Oligocene gekkotans include *Cadurcogekko* from localities within the Phosphorites du Quercy, France [9,33,37]. Two species of this genus are currently regarded as valid: the type species, *C. piveteaui* [37] and *C. verus* [38] (late Eocene MP 17 [38]). Note that *Cadurcogekko rugosus* [9] has recently been reidentified as a scincid and accordingly placed in its own genus, *Gekkomimus* [38]. Recently, new well-preserved material of *C.* cf. *piveteaui* has been described from the Phosphorites du Quercy [33].

### 1.1. Institutional abbreviations

CAS, California Academy of Sciences; IRSNB R, Institut royal des Sciences naturelles de Belgique, Fossil Reptile collection; MCZ, Museum of Comparative Zoology, Harvard University, Cambridge, MA, USA.

## 2. Material and methods

The studied material is housed in the Royal Belgian Institute of Natural Sciences, prefixed under individual IRSNB R numbers. The fossil specimens were photographed using a Leica M125 binocular microscope with an axially mounted DFC500 camera (LAS software (Leica Application Suite) v. 4.1.0 (build 1264)) and imaged on nano-CT using the micro-CT facility at the Slovak Academy of Sciences in Banská Bystrica, using a Phoenix mikro-CTv|tome|x L240. The specimen IRSNB R 456 was imagined on a RX Solutions EasyTom 150 at Institut royal des Sciences naturelles de Belgique. The CT datasets were analysed using VG Studio Max 3.1. and Avizo 8.1. Some comparative extant specimens were imaged with micro-CT at the University of Texas High-Resolution X-ray CT Facility and Harvard University Center for Nanoscale Systems (CNS).

## 3. Geological setting

The remains of the Dormaal gekkotans (except IRSNB R 456) were discovered by one of us (RS) in 1990 at the Dormaal stratotype, which is in the municipality of Zoutleeuw, in eastern Belgium (figure 1). The Dormaal Sand Member consists of a series of thin and discontinuous layers of fluviatile pebbles, cross-bedded lignitic and clayey sands with thin grey clay lenses pointing to rapidly changing deposition conditions in a fluviatile system [39,40]. It belongs to the lower part of the fluvio-lagoonal Tienen Formation that recorded the CIE of the PETM and contains abundant remains of terrestrial mammals and lizards, freshwater fish, chelonians and crocodylians. The Dormaal fauna, which represents the reference-level MP7 (BiochroM'97 [2]), has already yielded numerous mammal taxa, including the earliest modern placental mammals of Europe [41–43].

### 3.1. Systematic palaeontology

Squamata Oppel, 1811 [44]

Gekkota Camp, 1923 [45]

*Dollogekko* gen. nov.

*Etymology.* The genus is named in recognition of the Belgian palaeontologist Louis Dollo (Lille, 7 December 1857–Brussels, 19 April 1931) and gekko from the Malay ‘gekoq’, onomatopoeic of the call of Tokay gecko (*Gekko gecko*) which serves as the common name to all limbed gekkotans and sometimes pygopods.

*Diagnosis.* As for *Dollogekko dormaalensis*, the only known species.

*Dollogekko dormaalensis* gen. et sp. nov.

Figure 2

**Figure 2.**
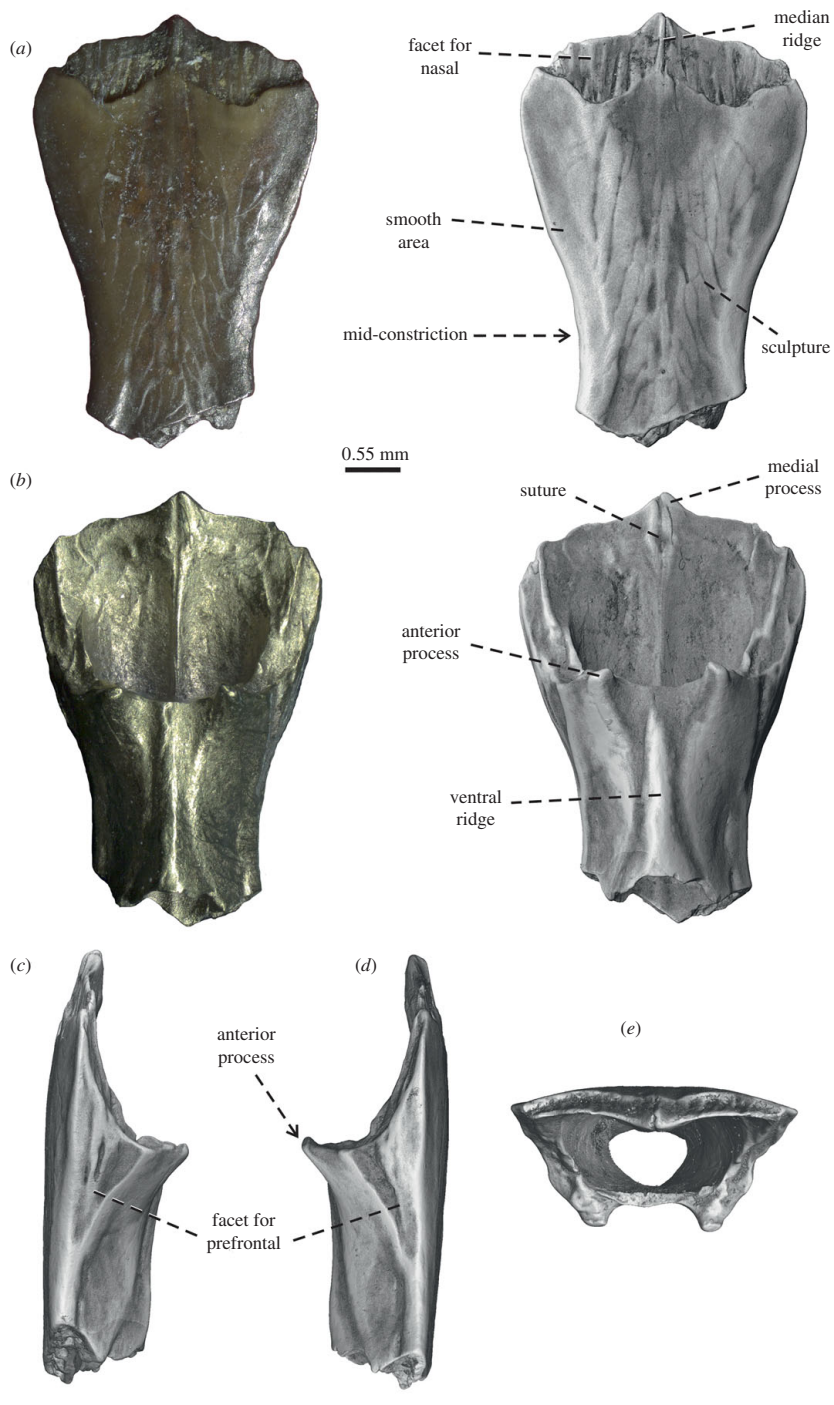
*Dollogekko dormaalensis* gen. et sp. nov., the holotypic frontal IRSNB R 452 in (*a*) dorsal, (*b*) ventral, (*c*) right lateral, (*d*) left lateral and (*e*) anterior views.

*Nomenclatural acts.* This publication and its nomenclatural acts are registered at ZooBank. LSID for this species: zoobank.org:pub:53515F23-394A-4DD3-B591-8EFD713C01A9

*Holotype.* The frontal bone IRSNB R 452.

*Locality and horizon.* Dormaal, Flemish Brabant, eastern Belgium, Dormaal member, Tienen Formation, Landen group, earliest Eocene (MP 7).

*Etymology.* The specific epithet *dormaalensis* is based on the locality of Dormaal in Belgium.

*Diagnosis.* A moderate-sized gecko (estimated SVL is approximately 70 mm) differing from all fossil and extant taxa of this clade in the following combination of features from the frontal: (i) anterior dorsal surface sculptured, flanks of the frontal or orbital margin smooth (versus smooth bones bearing no sculpturing in most living geckos, e.g. *Sphaerodactylus* and *Euleptes.* Note that the dorsal surface appears to be smooth in the Eocene *Geiseleptes delfinoi*, although a very short and shallow groove is visible extending along the lateral margin of the distal portion of each posterolateral process); (ii) the sculpturing has a faint grooved texture (*sensu* Glynne *et al*. [46]) (contra *Laonogekko lefevrei* and *Cadurcogekko piveteaui* in which furrows are generally deeper); (iii) grooves, which form the sculpturing, are short, sometimes interconnected and oriented slightly diagonally, from anteromedial to posterolateral (contra *Laonogekko lefevrei* and *Cadurcogekko piveteaui*, in which a series of longitudinal grooves in the anterior part transition to a network with some anastomosis towards the centre of the bone; contra *Rhodanogekko vireti* in which bumps are present on the dorsal surface; contra pitted sculpture in extant *Blaesodactylus antongilensis* and *Chondrodactylus bibronii*; contra rugose sculpture in e.g. *Carphodactylus laevis*, *Phyllurus platurus* and *Saltuarius salebrosus*); (iv) moderate interorbital constriction—the widest anterior point is 1.75 times wider than the narrowest point of the interorbital constriction (in *L. lefevrei* and *C. piveteaui*, the constriction is less developed and the frontals are very wide; contra *Rhodanogekko vireti* with the highly constricted interorbital portion of the narrow frontal); (v) the frontal cranial crests bear two short anterior processes (contra *L. lefevrei*); (vi) the anterior region of the frontal has a well-developed triangular medial process, whereas the lateral processes are almost absent (contra *R. vireti* in which the medial process is absent; contra extant *Chondrodactylus* in which the lateral processes are slightly longer than the median process); (vii) well-developed nasal shelf, with a W-shaped nasofrontal seam; (viii) well-developed triangular lateral facet for the prefrontal, without creating an evident indentation (i.e. anterolateral margin smooth).

## 4. Description

### 4.1. Frontal

The anterior more-or-less half of the frontal is well preserved (figure 2), whereas its posterior portion, including the posteroventral processes, is broken off. The frontal is unpaired, and tubular with fused subolfactory processes, and an anteroposterior length of the preserved portion being 4.3 mm. The preserved portion includes the complete anterior end and extends to about ¾ of the orbit (figure 2*a,b*). The preserved portion is funnel shaped, with a moderate interorbital mid-constriction (the widest anterior point is 1.75 times wider than the narrowest point of the interorbital constriction—when complete, the frontal would have been hourglass-shaped). The lateral margins are rather concave in dorsal (and ventral) view and the whole element gradually widens anteriorly. Thus, the narrowest part of the frontal is in the middle of the bone at the constriction between the orbits. The lateral margins flare out onto the anterolateral corners. These corners form rounded bulges on each side of the bone. On the lateroventral margin of the orbital margins, there are large triangular facets for the prefrontals. Each facet is mainly visible in lateral view (figure 2*c*,*d*), and its surface is rough. The prefrontal facet is triangular, reaching about half the length of the fused subolfactory process (see below). However, the lateral margins of the frontal are not indented, due to the presence of these facets in dorsal view. The overall dorsal surface of the frontal is flat and faintly sculptured, having a wrinkled appearance. The ornamentation pattern consists of oblique grooves delimiting flat, semi-oblong areas among them. The grooves are mainly concentrated in the middle of the bone, whereas the flanges tend to be smooth and unsculptured. The grooves have an anteromedial–posterolateral orientation (in contrast with a network with some anastomosis towards the centre of the bone in *Laonogekko lefevrei* and *Cadurcogekko piveteaui*). There is a faint ridge in the midline that might represent the fusion line between left and right halves; this is most pronounced anteriorly and fades away posteriorly. In the anteriormost region, two well-demarcated shelves for the nasals are visible. The surface of each shelf bears numerous longitudinal ridges, and the shelves are partly separated by the median ridge. These facets' posterior margins have a wavy appearance, defining a rounded ‘W’. The anterior margin of the frontal has a small blunt projection; this medial process is clearly longer than the lateral processes of the frontal, which are almost absent. When the nasals were articulated, three subequal and more superfical projections were defined, two anterolateral and a medial projection.

Ventrally, the frontal cranial crests are well-developed, forming subolfactory processes that are completely fused ventrally to form a tubular bone (figure 2*e*). The anterior and posterior ends of the tube are preserved. There is a pronounced ventral ridge along the midline of the subolfactory processes, forming a low keel. Anterior to the subolfactory processes, the cranial crests extend downward and are capped by two peg-like anterior processes. There is a flattened ventral surface on each side of the ‘keel’. The two peg-like anterior processes are joined medially by a slightly concave margin. The tubular frontal is wider anteriorly than posteriorly (being more funnel-like than tunnel-like), also indicating that the olfactory tracts were expanded anteriorly, closer to the nasal cavity and vomeronasal organ.

Gekkota indet. 1

Figure 3 (*a*–*h*)

**Figure 3.**
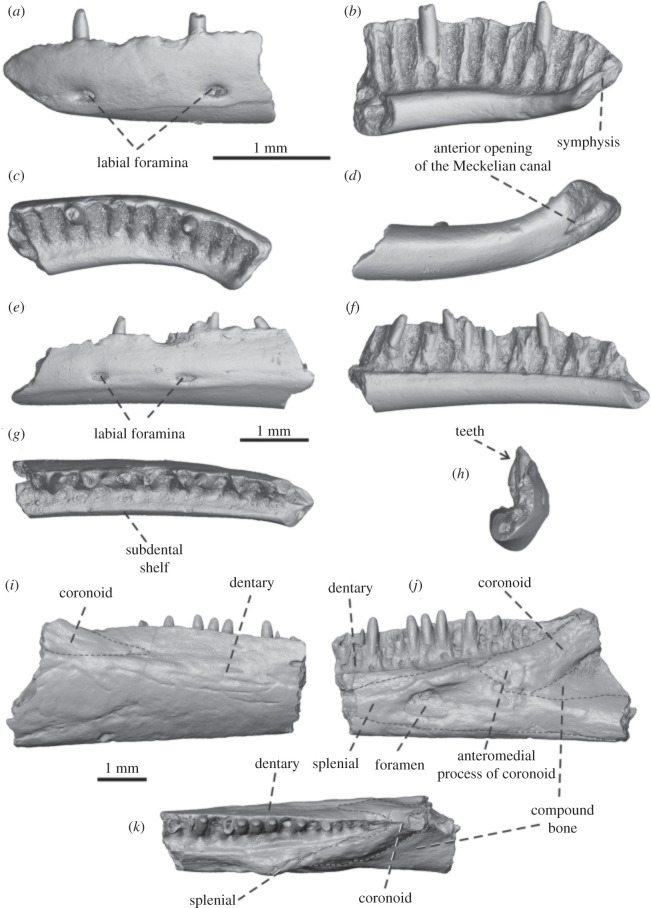
Gekkota indet. 1, the dentaries IRSNB R 454 and IRSNB R 453; Gekkota indet. 2, the mandible fragment IRSNB R 456 in (*a*,*e*,*i*) lateral, (*b*,*f*,*j*) medial, (*c*,*g*,*k*) dorsal, (*d*) ventral and (*h*) anterior views.

*Material.* Two left dentary fragments IRSNB R 453 and IRSNB R 454.

*Locality and horizon.* Dormaal, Flemish Brabant, eastern Belgium, Dormaal member, Tienen Formation, Landen group, earliest Eocene (MP 7).

### 4.2. Dentary

Two fragments of left dentaries, associated with the frontal, are preserved (figure 3*a–h*). Specimen IRSNB R 454 represents the anteriormost portion of the dentary (figure 3*a–d*). It has a small symphysis and possesses 11 tooth loci (two teeth are still attached, one with a complete rounded crown and the other with a worn crown). Specimen IRSNB R 453 represents the middle to anterior portion of the dentary (figure 3*e–h*), but the anteriormost portion is missing. This specimen possesses 13 tooth loci (three teeth still have the crown, and six root fragments of others are still attached; note the total tooth number was certainly much higher, because the posterior region is missing). Based on these fragments, it can be estimated that the dentary, when complete, was a long and slender bone, and had a completely fused Meckelian canal, as in crown gekkotans, contrary to the Cretaceous gekkonomorph *Hoburogekko suchanovi*, in which the Meckelian canal is partially fused [47,48]. In dorsal view, the anteriormost part of the symphyseal region is slightly curved (figure 3*c,d*). This anatomy of the mandibular symphysis might have produced the characteristic inverted ‘V’ shape typical of geckos [49]. Here, a short anterior opening of the Meckelian canal is visible in the ventral view (figure 3*d*). The alveolar crest (bearing the teeth) is tall, much higher than the ventral portion of the dentary. The subdental shelf is moderately expanded medially, forming a clearly visible surface in the dorsal view. The otherwise smooth external surfaces of both specimens are pierced by two elliptical labial foramina (figure 3*a*,*e*).

*Dentition:* The teeth are closely spaced and have a pleurodont implantation, with deep roots, although not as deep as in iguanians. The teeth are tall, overarching the dental crest by ⅓ of the tooth length. They are conical, slender, straight and distinctly pointed with narrow interdental spacing.

Gekkota indet. 2

Figure 3(*i*–*k*)

*Material.* The fragment of mandible IRSNB R 456.

*Locality and horizon.* Dormaal, Flemish Brabant, eastern Belgium, Dormaal member, Tienen Formation, Landen group, earliest Eocene (MP 7).

### 4.3. Dentary

Specimen IRSNB R 456 represents a fragment of the right mandible (figure 3*i–k*). It is relatively robust and distinctly dorsoventrally high. Besides the posterior portion of the dentary, it also includes the anterior portion of the coronoid and most of the splenial. The dentary is tubular. The sutures among the bones are faint, and the CT scan does not permit every individual bone to be isolated; however, it appears that the coronoid abutted the posterodorsal side of the splenial. The dentary preserves fragments of eight teeth, but there are 16 visible loci. The splenial has the characteristic triangular shape and is broken in the middle, where the anterior dental alveolar foramen should be. A faint indication of the anterior mylohyoid foramen is visible between the splenial and the dentary. The anterior portion of the coronoid is preserved and clasps the dentary laterally and medially—the anteromedial and anterolateral processes are clearly recognizable. The coronoid eminence was probably tall, unlike the very reduced one of miniaturized geckos [50].

*Dentition:* Tooth attachment is pleurodont and tooth replacement occurs lingually. Teeth are conical, with rounded crowns and bases that have nearly parallel sides. The apex presents a labial and a lingual cusp separated by a shallow but well-visible anteroposterior gutter (visible on all the teeth except the last preserved one).

## 5. Discussion

Lizards from the Dormaal locality were only briefly discussed by Hecht & Hoffstetter [51] and, moreover, the specimens were not figured by these authors. Besides this, few other squamate specimens from this Belgian locality have been described [11,52–56]. The new gekkotan described here is an additional and important contribution to the site's squamate assemblage. The frontal and dentaries described here can be referred to Gekkota on the basis of the following characteristics [21,23,49]: (i) absence of osteoderms fused to the skull bones; (ii) fused frontal; (iii) fused subolfactory processes of frontal in the midline to form a funnel-like structure; (iv) dentary with a closed Meckelian canal, and the high number of conical pleurodont teeth; (v) inverted ‘V’ shaped mandibular symphysis (implied); and (vi) a coronoid that clasps the dentary laterally and medially.

**Figure 4.**
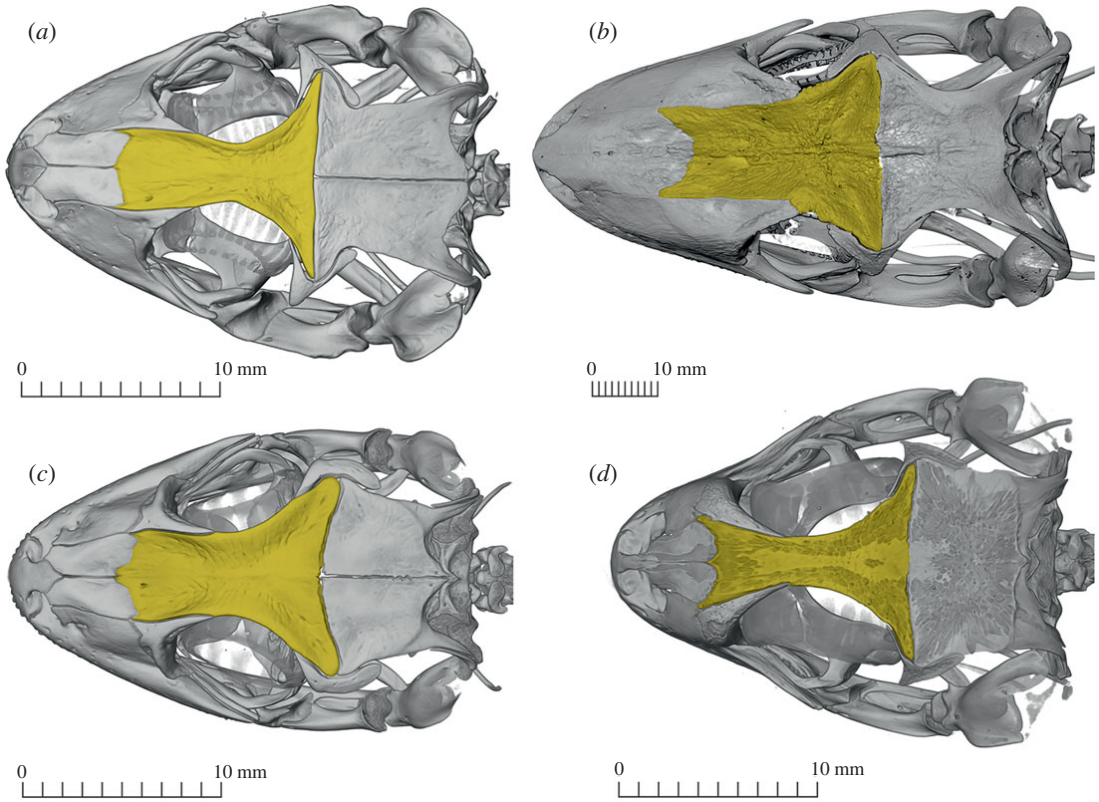
Dorsal view of the skull of some extant geckos exhibiting diversity of frontal bone shape and sculpturing (yellow). (*a*) Carphodactylidae, *Underwoodisaurus milii* (CAS 74744), (*b*) Diplodactylidae, *Rhacodactylus leachianus* (MCZ–R15967), (*c*) Phyllodactylidae, *Thecadactylus rapicauda* (CAS 95146) and (*d*) Gekkonidae, *Chondrodactylus angulifer* (CAS 126466).

The Dormaal frontal shows several unique features, such as the sculpture pattern with its orientation of the grooves as well as its general shape, and the shape and size of the articular facets with the nasals and prefrontals (see Diagnosis and Description). On this basis, we have decided to erect the new genus and species name *Dollogekko dormaalensis*. In addition to the fossil gekkotans *Laonogekko lefevrei* and *Cadurcogekko*, grooved sculptured frontals are also known in modern representatives of every gekkotan family [46] (figure 4): Carphodactylidae (*Underwoodisaurus milii*, *Nephrurus levis*), Diplodactylidae (*Rhacodactylus laechianus*, *Hoplodactylus duvaucelii*, *Lucasium damaeum*), Eublepharidae (*Eublepharis macularius*, *Holodactylus africanus*, although sculpturing in these taxa is very superficial and almost smooth), Sphaerodactylidae (*Quedenfeldtia trachyblepharus*), Phyllodactylidae (*Heamodracon riebeckii*, *Thecadactylus rapicauda*, *Phyllopezus lutzae*) and Gekkonidae (*Chondrodactylus angulifer*, *Hemidactylus turcicus*; see also Villa *et al*. [57]). However, the combination of faint sculpturing and frontal shape is unique to *Dollogekko dormaalensis*.

Considering the size alone, the two dentary fragments referred to here as Gekkota indet. 1 are comparable in depth of the medial wall, tooth size, dentition spacing, tooth density (about five teeth in 1 mm), size and shape of the labial foramina, and position of these foramina (just above the labial ridge). Although the curvature of the dentaries looks different (curved in one, and straighter in the other), this could be due to the fact that both fragments preserve different sections of the bone, one is the anterior portion including the symphysis, and the other fragment, the mid-portion of the dentary. Whether the dentary fragments and the frontal bone can be assigned to the same taxon is a more difficult subject. Since the material was recovered from the same locality by one person (RS) in a single pit during the 1990 excavation and the proportions of these elements are consistent with the idea that these bones correspond to a gekkotan of about the same size, one alternative would be to include the two anterior fragments of the dentary in the same taxon. At Dormaal, most modern clades make their first occurrence in the European fossil record and are represented by a single species each. In our case, however, there are not many diagnostic characters that can be correlated between these elements. Dermal sculpturing in theory could be used to correlate these bones, but in a recent survey of gekkotans that included a sample of 18 gekkotans with grooved sculptured frontals, only one (*Homopholis walbergii*) had grooved sculptured dentaries, while the remaining 17 species had smooth dentaries [46]. Unfortunately, smooth-surfaced bone is the widespread condition in Gekkotans [58] and cannot be used as a strong argument for association of these elements.

The third fragment of the jaw available from this locality, which was originally mentioned by Augé [9], is particularly interesting. Besides the dentary, it possesses at least a coronid and a splenial. Such a preservation is unique for European gekkotan fossils. This specimen clearly represents a different taxon from *Dollogekko*, and this can be easily determined using its large size alone. The frontal of *Dollogekko* is from a skeletally mature individual, revealed by the fusion of the two halves that made this bone (mostly fused in skeletally mature gekkotans, but separated in juveniles and a few paedomorphic forms (e.g. *Geckolepis*, *Lygodactylus*, *Phelsuma*, *Coleodactylus*, *Teratosincus* and the gekkonomorph *Norellius nyctisaurops*) [59–61]. So it is possible that the frontal was near the maximun size; the fragment of the jaw is about twice as large as the other two dentary fragments. For this reason, the mandibular fragment is referred to Gekkota indet. 2. This is an evidence that at least two gekkotan species occurred in Dormaal.

The material from Dormaal, with its age of around 56 Myr, together with a material of indeterminate gekkotan reported from Silveirinha [10], represents the earliest Cenozoic representatives of these lizards in Europe. *Dollogekko*, together with the 5 Myr younger (MP 10) *Laonogekko lefevrei* from France [36], formed an early Palaeogene radiation of this clade. The palaeoclimatic conditions during the Eocene in Northern Europe were highly suitable for a diverse subtropical to tropical herpetofauna. This fauna included representatives of squamate groups (e.g. iguanians) that no longer survive in the region [9,25,53]. Today, members of Gekkota are distributed worldwide in warm temperate to tropical areas [13,62], although some species reach temperate regions in Asia, Patagonia and New Zealand [62–64]. In Europe, *Euleptes*, *Tarentola*, *Hemidactylus* and *Mediodactylus* are today distributed only in the Mediterranean region, whereas Lacertidae is Europe's dominant group of lizards, occurring even above 60° North latitude in places [14,65]. *Dollogekko* certainly represents a thermophilic element in Dormaal. The PETM at the time of the earliest Eocene allowed the distribution of this lizard group in this northern latitude (above 50° North—the level of the southern part of England). This is also supported by the occurrence in amber of *Yantarogekko* from the Kaliningrad District (latitude: 54.7°). Although the comparison of *Dollogekko* to *Yantarogekko* is not possible due to their different modes of preservation, it is very unlikely that the Dormaal material represents the latter taxon. *Dollogekko* is most likely geologically much older (at least 10–15 Myr; see Introduction). Moreover, all the Baltic amber subunits are considered to have originated from territories of the Fennosarmatia landmass [66]. This area was isolated from the area of Dormaal, because the increase in temperatures during the early Eocene led to a rise in sea level, and many areas of Eurasia were submerged. Europe was an archipelago comprising various islands [67] and the Fennosarmatia landmass and the area of Dormaal formed different parts of this European archipelago. Nonetheless, together these finds demonstrate the expansive northern distribution of gekkotans in Europe during the Eocene. The importance of understanding past distribution of thermophilic (tropical) species during this geological epoch is magnified in light of future global climate change and potential future changes in sea levels, as well as the distributions of certain infectious diseases, e.g. malaria [68,69]. Note: Dr. Marc Augé confirmed that there is no mention of gekkotans from Dormaal in his 1990 paper [11]; however, he pointed out that the anterior part of a gekkotan frontal was illustrated previously in Augé [70], fig. 1L. Although neither formal description of this frontal bone nor information about its repository or specimen number was provided with this figure, there is no doubt that it is the holotype of *Dollogekko* described by us, thus Augé [70] constitutes the first published record of this material.

## Data Availability

All specimens are catalogued and accessible in the fossil reptile collection of the Institut royal des Sciences naturelles de Belgique, Brussels. Digital surface models of the figured fossil specimens IRSNB R 452-454 are available on Morphosource and Virtual Collections: IRSNB R 452: https://www.morphosource.org/concern/media/000433880?locale=en. IRSNB R 453: https://www.morphosource.org/concern/media/000433889?locale=en. IRSNB R 454: https://www.morphosource.org/concern/media/000433902?locale=en IRSNB R 456: https://www.morphosource.org/concern/media/000446189?locale=en.
